# Transcriptome Response of Atlantic Salmon (*Salmo salar*) to a New Piscine Orthomyxovirus

**DOI:** 10.3390/pathogens9100807

**Published:** 2020-09-30

**Authors:** Francisca Samsing, Pamela Alexandre, Megan Rigby, Richard S. Taylor, Roger Chong, James W. Wynne

**Affiliations:** 1 CSIRO Agriculture and Food, Hobart 7004, Australia; Megan.Rigby@csiro.au (M.R.); Richard.Taylor@csiro.au (R.S.T.); 2 CSIRO Agriculture and Food, Brisbane 2601, Australia; Pamela.Alexandre@csiro.au (P.A.); Roger.Chong@csiro.au (R.C.)

**Keywords:** host–pathogen interactions, interferon, T cell-mediated immunity, Pilchard orthomyxovirus (POMV)

## Abstract

Pilchard orthomyxovirus (POMV) is an emerging pathogen of concern to the salmon industry in Australia. To explore the molecular events that underpin POMV infection, we challenged Atlantic salmon (*Salmo salar*) post-smolts in seawater via cohabitation. Tissue samples of the head kidney and liver were collected from moribund and surviving individuals and analyzed using transcriptome sequencing. Viral loads were higher in the head kidney compared to the liver, yet the liver presented more upregulated genes. Fish infected with POMV showed a strong innate immune response that included the upregulation of pathogen recognition receptors such as RIG-I and Toll-like receptors as well as the induction of interferon-stimulated genes (*MX, ISG15*). Moribund fish also presented a dramatic induction of pro-inflammatory cytokines, contributing to severe tissue damage and morbidity. An induction of major histocompatibility complex (MHC) class I genes (*B2M*) and markers of T cell-mediated immunity (*CD8-alpha, CD8-beta, Perforin-1, Granzyme-A*) was observed in both moribund fish and survivors. In addition, differential connectivity analysis showed that three key regulators (*RELA/p65*, *PRDM1,* and *HLF*) related to cell-mediated immunity had significant differences in connectivity in “clinically healthy” versus “clinically affected” or moribund fish. Collectively, our results show that T cell-mediated immunity plays a central role in the response of Atlantic salmon to the infection with POMV.

## 1. Introduction

Pilchard orthomyxovirus (POMV) is a virus of concern to the Atlantic salmon industry in Tasmania, Australia [1]. The virus was first isolated in 1998 as an incidental discovery from wild pilchards (*Sardinops sagax*) collected from the waters off the coast of South Australia [1]. Later in 2006, a similar orthomyxo-like virus was isolated from farmed Atlantic salmon in Tasmania during routine surveillance. No further orthomyxo-like viruses were isolated from farmed Atlantic salmon until 2012, when a serious outbreak causing the mortality of more than 500,000 fish was detected in south-eastern Tasmania. The orthomyxoviruses isolated from pilchards and farmed Atlantic salmon were confirmed to be the same virus [1]. POMV belongs to the *Orthomyxoviridae* family, which includes serious human and animal pathogens such as influenza A viruses (IAV) and infectious salmon anaemia virus (ISAV), which is a notifiable finfish disease under the regulations of the World Organization for Animal Health, Office International des Epizooties (OIE) [2]. The clinical disease caused by POMV, recently named salmon orthomyxoviral necrosis (SON) [3], causes lethargy, dark coloration of the skin, and petechial hemorrhages on the ventral areas of the body. Unlike infectious salmon anaemia (ISA), the disease caused by ISAV, POMV does not cause anemia on its host. In addition, the in vivo manifestation of SON in farmed Atlantic salmon appears to occur more rapidly than ISA [3]. Histopathological findings in clinically affected fish include cellular necrosis in different organs, such as the liver, spleen, kidneys, and heart. Previous studies have characterized the virus and developed effective diagnostic tools to detect fish showing clinical signs of POMV based on real-time PCR [1]. However, further research is required to gain a better understanding of the host immune response to the disease and its pathogenesis at a molecular level to ultimately reduce virus-induced mortalities.

POMV is a single-stranded RNA virus with eight viral segments encoding ten putative proteins. Phylogenetically, ISAV is the closest related virus, but a comparison of the six major proteins encoded by ISAV and POMV provide strong evidence that these orthomyxoviruses are quite divergent [1]. The organization of the POMV genome appears similar to ISAV, but they share less than 40% amino acid sequence homology in the PB1 subunit of the viral RNA polymerase [1], which is generally the most conserved segment in orthomyxoviruses. Despite their genomic divergence, a recent transcriptomic study using RNA sequencing (RNA-seq) to compare the responses of Atlantic salmon kidney (ASK) cells to the infection with POMV and ISAV revealed that both pathogens induced significant and similar innate antiviral responses [4]. Early upregulation of pathogen recognition receptor genes, RIG-I and toll-like receptors (TLR3), was observed in response to both viruses and triggered downstream interferon (IFN) responses. Interferon-stimulated genes (ISGs), including *MX*, *ISG15* and *Viperin*, were significantly upregulated in the early stages of infection with both viruses. It is generally accepted that key proteins of the type I IFN system are induced during the infection with ISAV, but these are unable to inhibit viral replication both in vitro [5] and in vivo [6,7]. Similar findings have been observed for POMV in vitro [4], but the transcriptomic response to POMV infection in vivo still requires further investigation.

High-throughput technologies to survey gene expression, such as RNA-seq, have enabled the study of host responses to disease at the level of whole transcriptome rather than individual transcripts. Standard pipelines to analyze these large datasets use differential expression (DE) analysis to identify genes with the most significant differences between contrasting conditions. However, differential expression analysis requires multiple testing corrections that can sometimes impede the discovery of genes with subtle differences in expression. A powerful approach to overcome this problem is gene co-expression network analysis [8]. Co-expression networks constructed from RNA expression data can effectively capture relationships between transcripts and reveal a biologically meaningful higher-order organization of the transcriptome [9].

The transcriptome response of hosts to the challenge with POMV has only been investigated in vitro [4]. To this end, here, we challenged Atlantic salmon post-smolts in seawater via cohabitation with POMV-injected fish to mimic a natural route of infection and explored the host responses to infection. Tissue samples of the head kidney and liver were collected from moribund and surviving individuals; then, they were analyzed using RNA-seq to characterize viral and host gene expression profiles and construct gene co-expression networks. Examining whole transcriptome sequencing and gene co-expression profiles of Atlantic salmon challenged with POMV will improve our understanding of the pathogenesis of this disease at the molecular level and may shed light on the protective mechanisms that underpin resistance.

## 2. Results

### 2.1. POMV Challenge and Pathology

Morbidity of intraperitoneally (IP)-injected fish (trojans) started four days post-infection (dpi) and peaked at 12 dpi, with a cumulative morbidity of 57.1%. Morbidity in the cohabitant fish commenced at 6 dpi and peaked at 18 dpi, with cumulative morbidity of 51.5%. All moribund fish (POMV-positives and POMV-suspects) presented clinical signs consistent with POMV pathology, including loss of equilibrium, lethargy, lack of response to stimuli, and dark pigmentation of the skin [3]. At the necropsy, moribund fish displayed generalized congestion, petechial hemorrhaging of visceral fat and internal organs, splenomegaly, and yellow fluid and mucus inside the gastrointestinal tract. These symptoms constituted our case definition for moribund fish affected by POMV. POMV-positive moribund fish were those that tested positive to the real-time PCR [1], with a cycle threshold (C_T_) value < 38 in both tissues (liver and head kidney), and POMV-suspect moribund fish were those with either no C_T_ or a C_T_ ≥ 38. Results will mainly focus on the response of POMV-positive moribund fish and survivors, which were fish that had been exposed to POMV but were still alive at the end of trial (20 dpi) and showed no clinical signs of disease. One out of three sampled survivors tested positive for POMV, and only one POMV-suspect had a negative real-time PCR in both tissues. A summary of all diagnostic results is presented in Supplementary Table S1.

**Figure 1:**
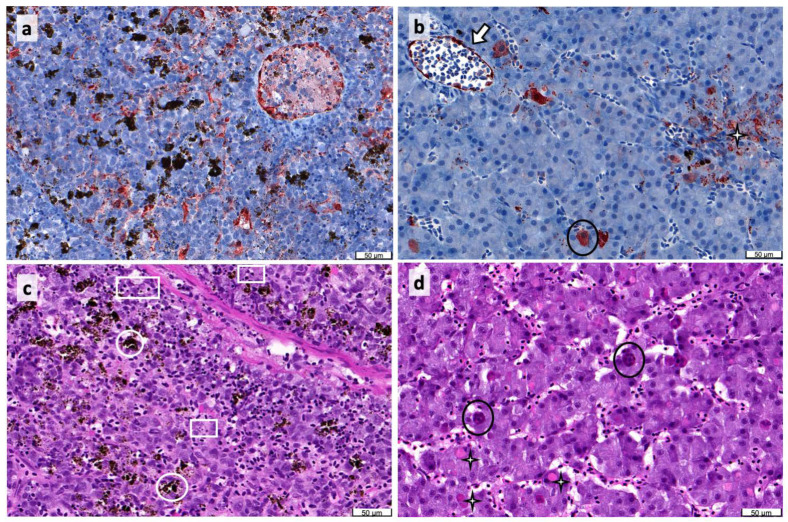
Micrographs of moribund Atlantic salmon (*Salmo salar*) infected with Pilchard orthomyxovirus (POMV): (**a**) Immunohistochemical staining of POMV in head kidney showing severe generalized POMV signals in many cells, including melanomacrophages, melanin-like granules, and a round cluster of reactive hematopoietic cells (top right corner); (**b**) Immunohistochemical staining of POMV in liver showing clusters of positive POMV melanomacrophages (star), positive apoptotic hepatocytes (circle), and positive endothelial cells (arrow); (**c**) Histological changes in head kidney (hematoxylin–eosin (H&E) staining) showing necrosis of hematopoietic tissue, abundant cell debris, and pyknotic or karyorrhectic nuclei. Pyknotic nuclei are the small, spherical condensed and black structures. When these damaged nuclei fragment in the karyorrhectic process, they produce varied-sized, smaller black structures (white rectangles). In contrast, melanin granules (white circles) are in tighter clusters; (**d**) Histological changes in liver (H&E staining) showing eosinophilic hepatocellular inclusions (stars) and multinucleated apoptotic cells (circles) that are large and rounded, but undergoing cellular degradation. All tissues come from moribund individuals that tested positive to the real-time PCR assay for POMV. Scale bar = 50 μL.

**Figure 2:**
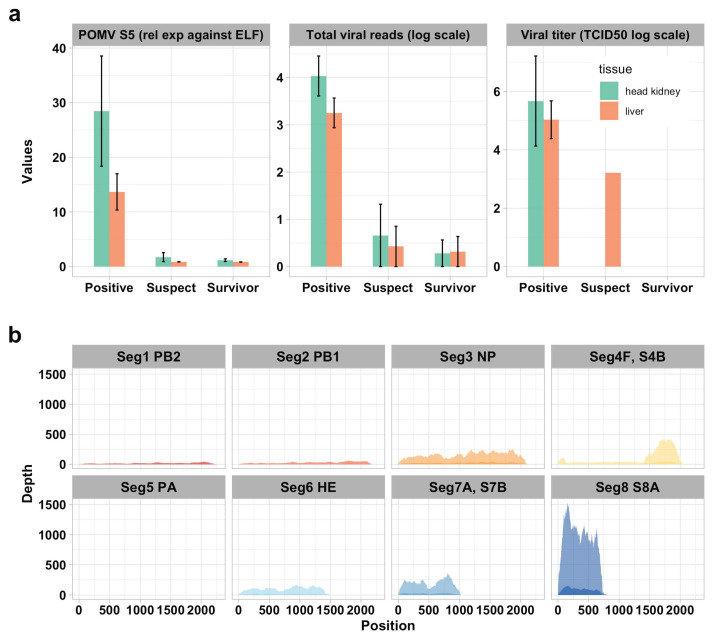
Pilchard orthomyxovirus (POMV) loads and viral gene expression profiles: (**a**) From left to right panels show mean relative POMV RNA per tissue using real-time PCR against elongation factor-1α (rel. exp. against ELF), total viral reads mapped to the POMV genome and virus titers in moribund POMV-positive fish (real-time PCR cycle threshold (CT) value < 38 in both tissues), moribund POMV-suspects (either no CT or a CT ≥ 38) and survivors (fish exposed to POMV, but still alive at the end of trial with no clinical signs of disease); (**b**) Expression of POMV genomic segments in moribund fish (lighter shade: POMV-positive fish, darker shade: POMV-suspect fish) mapping RNA-seq raw reads against the POMV genome. Read depth was calculated as mean per-base coverage, normalized by the proportion of viral reads among total reads in each sample. For conciseness, POMV-positive and POMV-suspect are refered to in the figure as *positive* and *suspect*, respectively. TCID50 = tissue culture infective dose 50%. A detailed description of the POMV genome structure is provided in Mohr et al. [1].

Infection with POMV in tissue sections was confirmed by immunohistochemestry (IHC) using a polyclonal anti-POMV immune serum (Figure 1), real-time PCR [1], and virus titration (Figure 2a). In POMV-positive fish, viral loads measured by real-time PCR, total reads mapped to the POMV genome, and viral titration were higher in the head kidney compared to the liver (Figure 2a), but differences were non-significant (independent *t*-tests, *p* > 0.05). In addition, per-base coverage of viral reads mapped to each segment of the POMV genome was almost 10-fold higher in the head kidney compared to the liver, but the transcription pattern of each segment was similar in both tissues (Figure 2b and Supplementary Figure S2).

Histopathological findings in moribund fish included the necrosis of haematopoietic cells in the head kidney (Figure 1c), with abundant cell debris and pyknotic nuclei (cells undergoing necrosis or apoptosis), and apoptosis of melanomacrophages. The melanomacrophages and their melanin granules were also positive to POMV. In the liver, hepatocytes presented cytoplasmic inclusion bodies, possibly viral inclusions, and apoptotic multinucleated cells undergoing cellular degradation. Liver also showed the degranulation of melanomacrophages. The overall inflammatory response was moderate—i.e., no notable infiltration with leucocytes, with the exception of the melanomacrophages. These may have a role in dampening the inflammatory response by releasing melanin granules that capture reactive oxygen species. Compared to fish sampled at earlier time points (data presented in Samsing et al. [10]), survivors presented a depletion of melanomacrophages in the head kidney.

### 2.2. POMV Gene Expression

Viral and host gene expression profiles were examined using transcriptome sequencing (RNA-seq) data. Read mapping to the viral genome revealed that all viral gene segments were expressed in infected tissues, but expression levels varied across different viral segments and tissues. Segments coding for the RNA polymerase complex (PB2, PB1, PA), namely segments 1, 2, and 5, showed lower expression compared to other segments (Figure 2b). In contrast, segments 4 (F and S4B), 7 (S7A and S7B), and 8 (S8A) had exceptionally high expression levels in moribund fish. The expression of individual viral segments was similar between the head kidney and liver, but the coverage of viral reads mapped to the POMV genome was higher in the head kidney. The number of reads mapped to the POMV genome as well as the total number of input reads are presented in Supplementary Table S1.

### 2.3. RNA Sequencing

**Figure 3:**
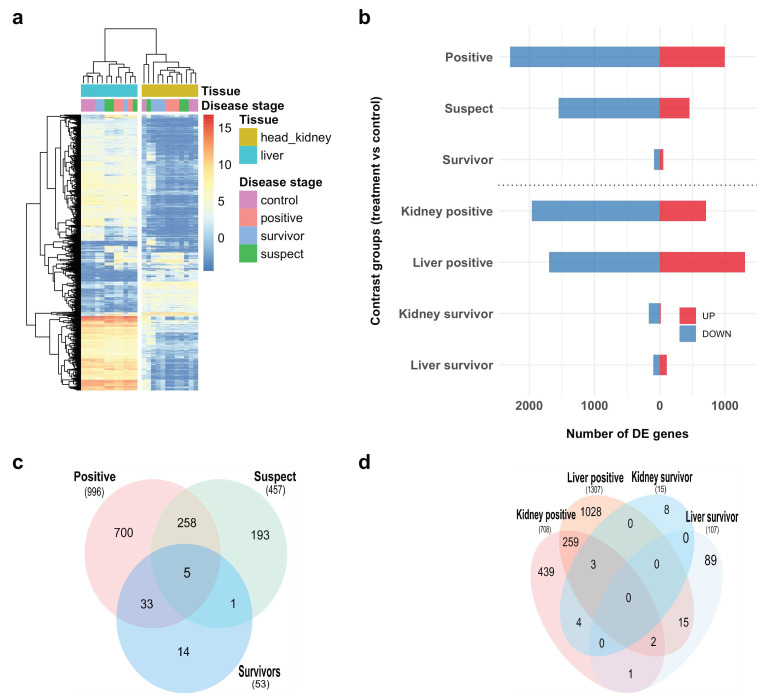
Host gene expression profiles of Atlantic salmon (*Salmo salar*) challenged with POMV: (**a**) Heatmap showing hierarchical clustering of normalized gene expression profiles for the top 1500 genes with the highest variance in liver and head kidney of control fish (fish sampled pre-challenged), moribund POMV-positive fish (real-time PCR cycle threshold (CT) value < 38 in both tissues), moribund POMV-suspect fish (either no CT or a CT ≥ 38) and survivors (fish exposed to POMV, but still alive at the end of trial with no clinical signs of disease); (**b**) Total number of differentially expressed (DE) genes, upregulated or downregulated compared to control fish grouped by disease stage (POMV-positive, POMV-suspect, survivor) in the top panel, or tissue type within disease stage in the bottom panel; (**c**,**d**) Venn diagrams of differentially expressed genes (upregulated with log2 fold change >2) compared to control fish, grouped by disease stage (**c**) and by tissue type within disease stage (**d**). For conciseness, POMV-positive and POMV-suspect are refered to in the figure as *positive* and *suspect*, respectively.

RNA-seq data were used to analyze host response to infection with POMV at different stages. Our mean alignment rate to the Atlantic salmon genome was 81.6% (≈21.9 million reads) corresponding to a mean of 36,946 (±679.6) assembled genes across samples. Read alignment statistics for each sample are provided in Supplementary Table S2. Prior to differential expression analysis, hierarchical clustering (Figure 3a), and multi-dimensional scaling (Supplementary Figure S1) showed that the transcriptome response was mainly driven by tissue type (liver/head kidney), followed by disease stage (moribunds/survivors).

### 2.4. Differential Expression and Functional Enrichment Analysis

Four groups were used in differential expression analysis: (1) POMV-positives, (2) POMV-suspects (both in a “clinically affected” or moribund stage), (3) survivors, and (4) uninfected controls (both 3 and 4 were in a “clinically healthy” stage). All comparisons were made against the control group. POMV-positive moribund fish had the largest number of differentially expressed (DE) genes followed by POMV-suspects and survivors in the model testing for the effect of disease stage (Figure 3b). The nested interaction model, which analyzed the effect of tissue type (liver/head kidney) within disease stage (only including POMV-positives and survivors) showed that liver had a higher number of upregulated genes compared to the head kidney in both POMV-positives and survivors. Most of the DE genes were downregulated at different disease stages and tissue types, regardless of the model used. The only exception was the liver of survivors, which had a higher number of upregulated genes. Tables with average expression and log2 fold change (logFC) values for all DE genes (adjusted *p*-value (Padj) < 0.05), and all contrasts are provided in Supplementary Tables S3 and S4.

**Figure 4:**
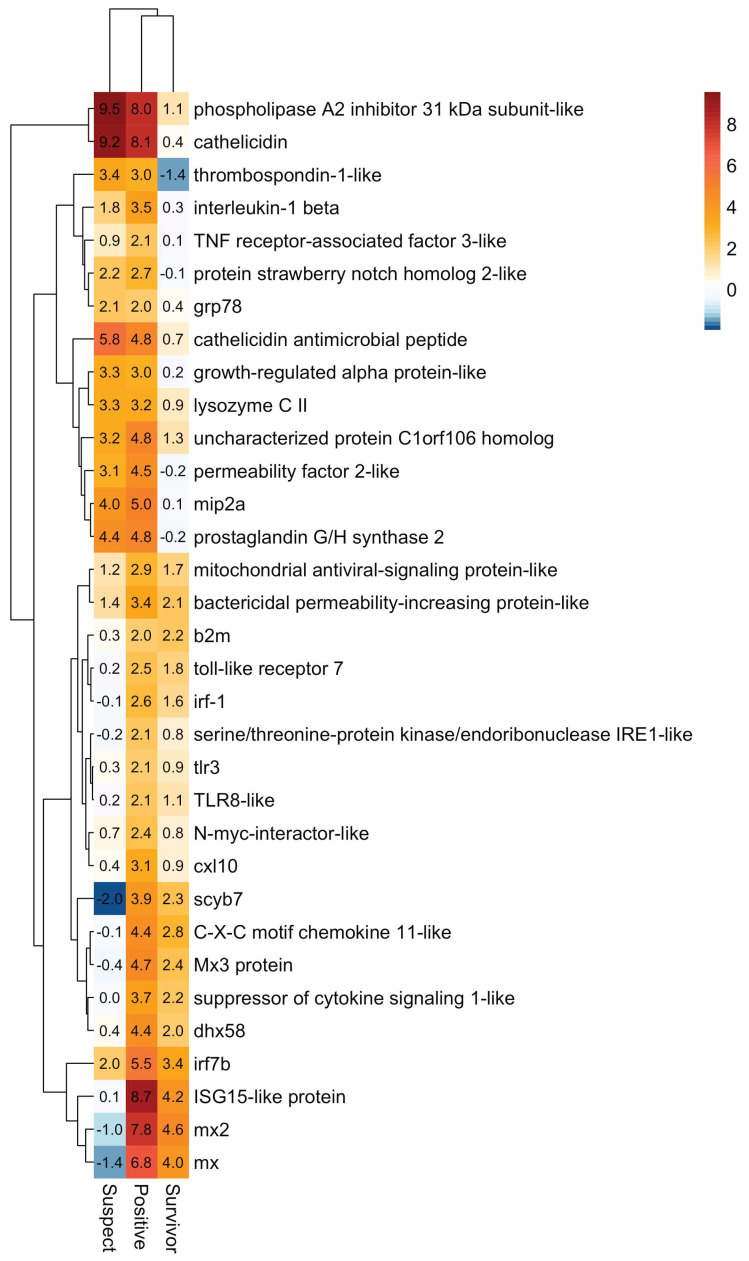
Heatmap showing genes involved in innate defense response (Gene Ontology term GO:0006952). Each tile represents the mean log2 fold change in regulation from three independent replicates in moribund POMV-positive fish (real-time PCR cycle threshold (CT) value < 38 in both tissues), moribund POMV-suspects (either no CT or a CT ≥8), and survivors (fish exposed to POMV, but they were still alive at the end of trial with no clinical signs of disease). For conciseness, POMV-positive and POMV-suspect are refered to in the figure as *positive* and *suspect*, respectively.

**Figure 5:**
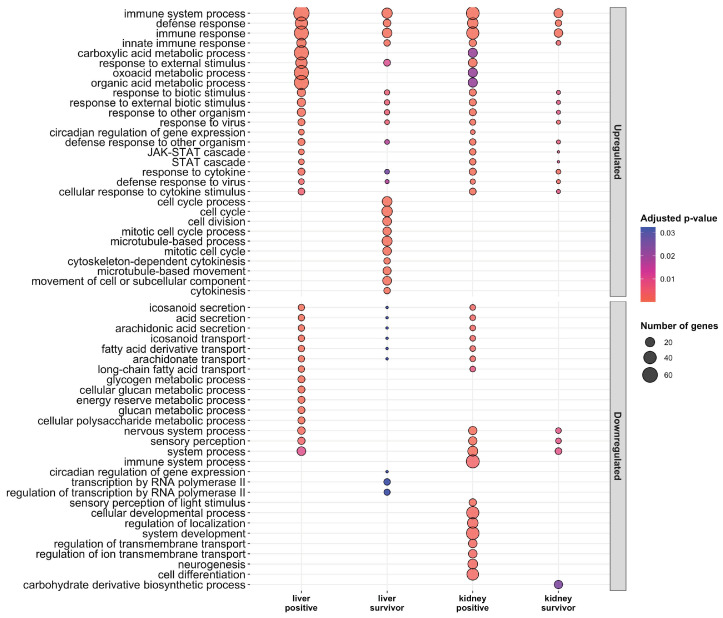
Gene Ontology (GO) enrichment analysis for upregulated and downregulated genes sets in the liver and head kidney of moribund POMV-positive fish (real-time PCR cycle threshold (CT) value < 38 in both tissues) and survivors (fish exposed to POMV, but still alive at the end of trial with no clinical signs of disease). For conciseness, POMV-positive and POMV-suspect are refered to in the figure as positive (liver positive and kidney positive) and suspect (liver suspect and kidney suspect), respectively.

Ranked lists of differentially expressed genes were used for functional enrichment analyses using Gene Ontology (GO) and KEGG databases. For the upregulated gene sets, significant GO enrichment was observed for immune response in all moribund fish (POMV-positives Padj = 2.9 × 10^−29^ and POMV-suspects Padj = 6.9 × 10^–6^). Genes in this pathway include interferon-stimulated genes (ISGs), such as MX 1-3, ISG15, IRF1, and IRF7, which were strongly induced in all stages, particularly in positive fish (Figure 4), with the highest levels of induction in the liver of positive fish. POMV-positive moribunds (Padj = 3.3 × 10^−2^) and survivors (Padj = 7.9 × 10^–8^) also showed an induction of genes involved in antigen processing and presentation (Figure 5), including MHC-class I genes, such as beta-2-microglobulin (B2M), and transport-associated proteins (TAP2a and TAP2b). Functional enrichment analysis for all groups is presented in Supplementary Table S5.

A strong enrichment of organic acid metabolic processes, specifically the carboxylic acid metabolic process (Figure 5), was observed in moribund fish (POMV-positives Padj = 3.8 × 10^−2^ and POMV-suspects Padj = 7.9 × 10^−4^), but not in survivors. Genes induced in this pathway included arginase 2 (ARG2), acyl-CoA synthetase short-chain family member 1 (ACSS1), 60 kDa lysophospholipase (LPP60), and a putative homolog of L-serine dehydratase/L-threonine deaminase (SDS-like, LOC106580394).

**Figure 6:**
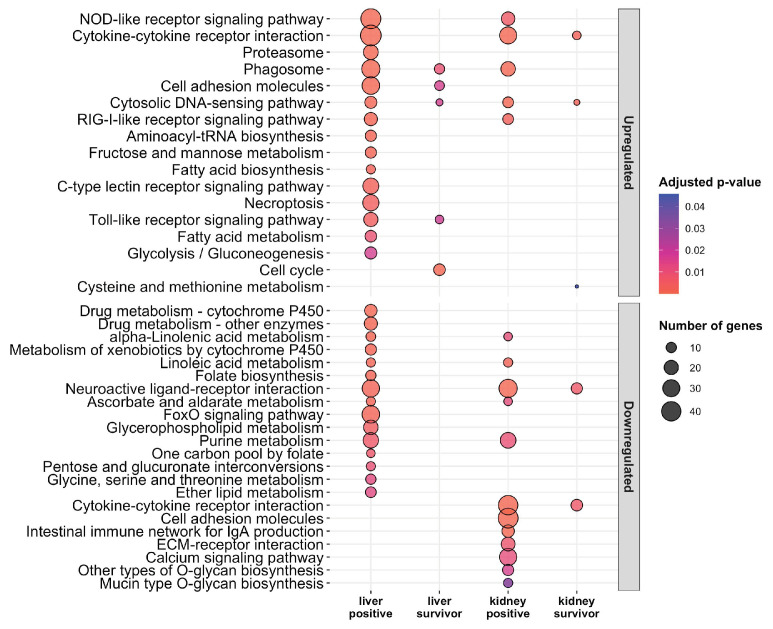
KEGG enrichment analysis for upregulated and downregulated genes sets in the liver and head kidney of moribund POMV-positive fish (real-time PCR cycle threshold (CT) value < 38 in both tissues) and survivors (fish exposed to POMV, but still alive at the end of trial with no clinical signs of disease). For conciseness, POMV-positive and POMV-suspect are refered to in the figure as positive and suspect, respectively.

**Figure 7:**
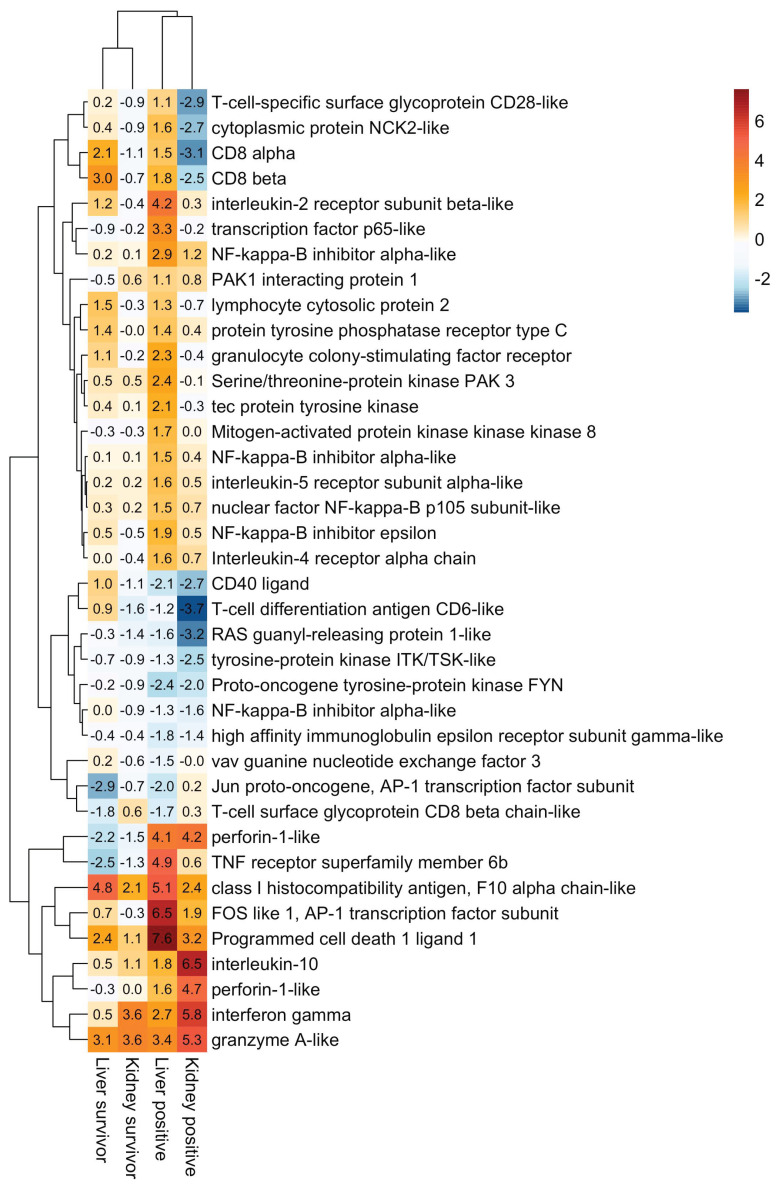
Heatmap of genes involved in T-cell receptor signaling pathway. Each tile represents the mean log2 fold-change in regulation from three independent replicates in the liver or head kidney of moribund POMV-positive fish (real-time PCR cycle threshold (CT) value < 38 in both tissues) and survivors (fish exposed to POMV, but still alive at the end of trial with no clinical signs of disease). For conciseness, POMV-positive and POMV-suspect are refered to in the figure as *positive* and *suspect*, respectively.

KEGG enrichment of POMV-positive moribunds showed an induction of classic innate antiviral recognition receptor pathways including RIG-I-like (Padj = 1.6 × 10^−4^), NOD-like (Padj = 1.6 × 10^−4^) and Toll-like receptor signalling pathways (Padj = 3.9 × 10^−5^) (Figure 6). The proteasome KEGG pathway, involved in antigen presentation, stress signalling, inflammatory response, and apoptosis, was also upregulated in POMV-positive moribunds (Padj = 2.3 × 10^−5^) but not in POMV-suspect moribunds. In the liver of POMV-positives, upregulated pathways included necroptosis (Padj = 3.6 × 10^−4^), activation of the phagosome (Padj = 5.1 × 10^−6^), and the cell adhesion molecules (CAMs) pathway (Padj = 6.2 × 10^−5^) (Figure 6). In contrast, the CAMs pathway was strongly downregulated in the kidney of POMV-positive moribunds (Padj = 3.8 × 10^−8^). This KEGG pathway includes multiple genes involved in T-cell signalling and adaptive immunity, such as CD6, CD8-alpha, CD8-beta, and CD28, which were downregulated in the kidney but strongly induced in the liver. In addition, genes involved in T cell-mediated cytotoxicity, such as Perforin-1-like genes (LOC106594078, LOC106595570) and Granzyme-A-like (LOC106570903) were also strongly expressed in POMV-positive fish (Figure 7).

### 2.5. Gene Co-Expression Network

Three networks were generated based on the same genes, namely: (1) ALL—considering all samples and tissues (n = 24), (2) HEALTHY—considering controls and survivors samples from both tissues (n = 12), and (3) MORIBUND—considering POMV-suspects and POMV-positives (n = 12). All three gene co-expression networks were composed of 1645 significant genes (nodes), which included 1297 (78%) DE genes, 366 (21%) key regulatory transcription factors, and 18 (1%) genes that were both DE genes and key regulatory transcription factors. In addition, 57% (n = 942) of these nodes had a higher average expression in head kidney, while the remaining nodes (43%, n = 703) had a higher average expression in liver.

Regarding the edges (or links) representing significant co-expression correlation, the ALL network had a total of 377,520 edges. The HEALTHY network had 90,777 (24%) edges with a stronger expression correlation in healthy fish (considering only genes with significant expression correlation ≥ |0.5|), and the MORIBUND network had 68,506 (18%) with a stronger expression correlation in moribund fish.

**Figure 8:**
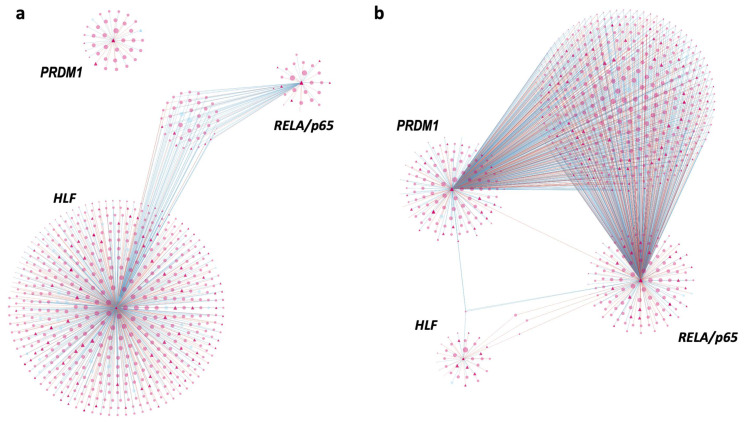
Gene co-expression networks constructed using the PCIT algorithm (see Section “Materials and Methods”) considering the first neighbors (direct connections) of the only three key regulatory transcription factors that were also differentially expressed (DE) genes and differentially connected by comparing the HEALTHY and MORIBUND networks. These three key regulatory factors included: *RELA* or transcription factor p65-like (*LOC106577543*), *PRDM1* or PR domain zinc finger protein 1-like (*LOC106588492*), and *HLF* or hepatic leukemia factor-like (*LOC106605810*). These networks were constructed using the following: (**a**) Edges or connections with higher co-expression correlation values in the HEALTHY network (considering controls and survivors); (**b**) Edges with higher correlation values in the MORIBUND network (considering POMV-positives and POMV-suspects). Both networks only consider genes connected by significant expression correlation values ≥ |0.5|. Nodes with a diamond shape correspond to transcription factors, circles represent DE genes, and blue circles are genes directly involved in innate immune functions. The size of the node is relative to the normalized mean expression values in the HEALTHY and MORIDUND networks, respectively. Edges are colored using a gradient from blue to red representing co-expression correlation values from −1 to 1 (only including values ≥ |0.5|). Annotated versions of these networks are provided as Supplementary Materials (Figures S3 and S4).

Differential connectivity analysis between HEALTHY and MORIBUND networks showed a total of 103 differentially connected nodes (genes): 31 were also differentially expressed (upregulated or downregulated in moribund fish), 69 were transcription factors, and three were both DE genes and transcription factors, suggesting that they play a central role as key regulatory factors during the infection with POMV. These three regulatory factors included: RELA or transcription factor p65-like (LOC106577543), PRDM1 or PR domain zinc finger protein 1-like (LOC106588492), and HLF or hepatic leukemia factor-like (LOC106605810) (Figure 8). Transcription factors RELA and PRDM1 were also within the top 10 most connected regulators in the MORIBUND network, with 550 and 542 connections, respectively (considering genes with significant expression correlation ≥ |0.5|). These key regulators also had higher expression values in moribund fish compared to healthy fish, and they were upregulated in both POMV-positives and POMV-suspects (RELA and PRDM1) and downregulated (RELA) or non-differentially expressed (PRDM1) in survivors. In contrast, HLF was within the topmost connected regulators in the HEALTHY network, with 597 connections (considering genes with significant expression correlation ≥ |0.5). This regulator also had a higher expression in healthy fish and was strongly downregulated in moribund individuals.

## 3. Discussion

This is the first transcriptome study profiling the response of Atlantic salmon (*Salmo salar*) to the infection with POMV in vivo. Fish in our trial were challenged in seawater with POMV by cohabitation with IP-injected animals to mimic a natural route of infection, as this can affect the innate immune response in salmon [11]. In this paper, we analyzed transcriptional responses during POMV infection in (1) clinically affected moribund fish, classified as POMV-positive or POMV-suspects, and (2) clinically heathy fish, which were either the control group or the survivors of the challenge. These groups were defined based on the overall transcription profile across samples, which indicated a similar gene expression pattern between POMV-positive and POMV-suspects, and control and survivors, particularly in the liver. We also evaluated the transcriptional patterns of expression of viral genes. Results from our experiment clearly show a significant difference in host gene expression profiles between tissues and within moribund and surviving individuals. Head kidney presented higher viral loads than liver in all infected animals, and although the expression pattern of viral genes was similar between tissues, the head kidney presented a greater read depth (more reads) mapped to each segment of the genome. Conversely, the liver presented more upregulated genes, which could be a reflex of the multiple functions of this tissue, including the defense response and metabolism of several complex molecules. Indeed, similar to previous results in vitro [4], POMV-positive fish showed a strong innate immune response that included the upregulation of pathogen recognition receptor (PRRs) such as RIG-I and Toll-like receptors (TLRs), and the induction of interferon (IFN)-stimulated genes. In addition, the initiation of an adaptive immune response that included antigen processing, presentation pathways, and cytotoxic T-cell immunity was observed in POMV-positive moribund and surviving fish.

Differences in transcriptome profiles were mainly driven by tissue, followed by disease stage. Hierarchical clustering between transcriptomes showed that the liver and head kidney could easily be separated into two major clusters (Figure 3a). Similar findings were observed in a transcriptome study of ISAV-infected Atlantic salmon, where major differences in expression profiles were detected between the gill, head kidney, and liver [12]. Similar to ISAV, POMV infection induced a larger number of differentially expressed genes in the liver compared to head kidney [6,12]. However, genes related to innate immune response, such as the type I IFN system, including IFN-stimulated genes and pathogen recognition receptors (RIG-I, TLRs), were upregulated in both tissues, as innate immunity is generally less tissue-specific.

Even though there was a strong induction of the innate immune system, this did not seem to provide protection against infection with POMV. The strong upregulation of IFN-alpha, IFN-induced genes (MX1-3, ISG15), and multiple pro-inflammatory cytokines and chemokines observed in POMV-positive moribund fish was seemingly unable to hamper viral replication given the high morbidity and tissue damage observed in our study. The dramatic activation of innate antiviral genes has also been observed in ISAV-infected cells [5,13] and fish [6], without conveying protection against viral infection. Unlike the infection with ISAV and POMV, interferon activity does confer protection to host cells against the infection with infectious pancreatic necrosis virus (IPNV), which is also an important pathogen of Atlantic salmon and a well-characterized RNA virus member of the *Birnaviridae* family belonging to the genus Aquabirnavirus.

One of the key advantages of RNA-sequencing is the simultaneous inspection of both host and viral gene expression profiles. Mapping of reads to the POMV genome indicated that all viral gene segments were expressed in infected tissues and had similar expression patterns in both liver and head kidney. This result is in contrast to previous findings of ISAV-infected fish [12], showing slightly different viral gene expressions profiles in different tissues [12]. In line with previous findings for POMV [4], segments coding for the RNA polymerase complex (PB2, PB1, PA) showed lower transcription values compared to other segments, namely segments 7 and 8. The high transcription of these viral segments compared during infection could be related to the function of the proteins they encode. The functional characterization of POMV proteins is still lacking and the POMV genome is fairly divergent from ISAV [1], but quite possibly, these proteins could have analogue functions to ISAV, including IFN-antagonizing activity [14].

Except for some pathogen recognition receptors and IFN-induced genes, there was little difference in gene transcription patterns between POMV-positives and POMV-suspects. Given that POMV-suspects were showing all the clinical signs associated with POMV, the most plausible scenario is that although these fish were infected, viral replication in the tissue had not yet reached detectable levels or samples were taken from sections of the tissue with lower viral loads. In addition, fish that tested negative to the real-time PCR assay could be in the viraemic phase, prior to replication in tissues. In support of this, two out of three sampled POMV-suspects succumbed earlier, around 7 to 8 dpi, in the course of the challenge experiment compared to most POMV-positive fish, which succumbed later, 12 to 15 dpi (Supplementary Table S1). Similar findings have been demonstrated in an ISAV challenge experiment [11], with salmon showing low viral loads in tissues during the early stages of infection, even though they have been exposed to the virus and were presenting clinical signs of disease. Moreover, a suite of pro-inflammatory cytokines, complement factors, and antimicrobial peptides was strongly induced in suspect fish, showing similar transcription levels to those in POMV-positive individuals.

A strong induction of pro-inflammatory cytokines and chemokines was observed in all moribund fish. The inflammatory response begins when the virus is recognized by pathogen recognition receptors, triggering the downstream release of specific pro-inflammatory cytokines, which enable organisms to respond promptly to infection. However, an excessive inflammatory response, can be the cause of further complications arising during viral infection. For example, disease severity associated with influenza A virus (IAV) is commonly associated with the hyperinduction of pro-inflammatory cytokines and insufficient control by the anti-inflammatory response – a combination of events called the ‘cytokine storm’ [15]. Interleukin-1β (IL-1β) was strongly upregulated in POMV-infected moribunds, suggesting a strong inflammatory response in these individuals. Interleukin-1β is one of the earliest expressed pro-inflammatory cytokines and regulates the expression of other cytokines and chemokines, such as IL-8. When administered directly into the intestine of grass carp (*Ctenopharyngodon idella*), IL-1β induces severe gut inflammation and the expression of TNF-α [16]. In mammals [17,18] and fish [19,20], the inflammatory response generated by IL-1β is tightly regulated by IL-10, whose function is to limit and, ultimately, terminate the immune response, preventing the damaging effects of inflammation and successful restoration of homeostasis [19]. In this study, IL-10 was strongly upregulated in moribund individuals, especially in the head kidney of POMV-positive fish (Figure 7), probably triggering the recruitment of melanomacrophages into the organ. These cells engaged specifically with POMV, but they may also play a role in dampening inflammation.

Survival of viral infection depends on the tolerance or endurance to high viral loads but also on the capacity of modulating inflammation, while still allowing an effective activation of the adaptive immune system. In this experiment, survivors presented enrichment for the antigen processing and presentation via the MHC class I pathway, with the significant upregulation of immunoglobulin-related genes such as beta-2-microglobulin (*B2M*) and class I histocompatibility antigen F10-α chain-like (*LOC106588401* or *UBA*) suggesting an activation of the adaptive immune system. Enrichment of this pathway was also observed in POMV-positive moribund fish, but early morbidity was probably associated to higher viral replication and a dramatic upregulation of innate immune mechanisms, leading to cellular stress and inflammation. Survivors, on the other hand, could have also presented a strong inflammatory response at the peak of infection, which they may have managed to moderate, permitting survival. However, these fish were sampled [20] after the start of the challenge, and with our current experimental design, it is not possible to determine whether they had such a response or not.

Unlike pro-inflammatory cytokines, chemokines have specific chemotactic activities that enable neutrophils and T-lymphocytes to migrate from blood vessels into the site of infection [21]. Here, POMV-positives and survivors showed an induction of chemoattractant chemokines that could be assisting with the recruitment of melanomacrophages. These included gene transcripts such as C-X-C motif chemokine 11 (*CXCL11*), which is also called *Interferon-inducible T-cell alpha chemoattractant (I-TAC)*, and macrophage inflammatory protein 2-alpha (*MIP2a*), which produces an interleukin-8 (IL-8) in *Salmo salar* (UniProt ID: B5XES8). The induction of IL-8 has also been observed in rainbow trout (*Oncorhynchus mykiss*) infected with viral hemorrhagic septicemia virus (VHSV) [22], which is a serious RNA virus affecting fresh and marine water fish, and Atlantic cod (*Gadus morhua*) head kidney cells stimulated with poly I:C [20], which is a synthetic analog of double-stranded RNA. One of the functions of IL-8 is to induce chemotaxis of granulocytes, primarily neutrophils, to the affected site to aid with viral clearance [23]. Infiltrating neutrophils also release chemokines (i.e., IL-8), which then reinforce the recruitment of additional neutrophils to the developing inflammation, perpetuating the inflammatory response [24]. The excessive recruitment and activation of neutrophils has been associated with immunopathology and lung tissue injury during the infection with IAV [25], but their role in viral clearance is still uncertain [26]. However, in our trial, we did not observe a large recruitment of neutrophils in moribund fish.

Induction of an effective cellular response has been correlated with survival and viral clearance in ISAV-infected Atlantic salmon [6,27]. The clearance of IAV is largely dependent on CD8+ T cells, which have to equilibrate control of the infection with immunopathology [28]. Cell damage in POMV-infected fish was evident in our histological observations, showing cell debris, pyknotic (condensed) nuclei, hepatocellular inclusions, and apoptosis. On the other hand, survivors showed an upregulation of cell cycle and cell division pathways indicating tissue repair, but they also presented a strong induction of *CD8-alpha* and *CD8-beta*, suggesting the activation of T cell-mediated immunity. Upon antigen recognition of viral epitopes presented on the cell surface by MHC class I molecules, which were also upregulated in survivors, cytotoxic T lymphocytes induce the apoptosis of target cells by releasing specialized lytic granules. These cytotoxic granules contain perforin, a pore-forming protein, and a group of cell-death inducing proteases called granzymes [29]. Perforin 1 was not induced in survivors, but it was strongly induced in both the liver and head kidney of POMV-positive moribunds. Nonetheless, granzyme A and interferon-gamma (IFNγ) were strongly expressed in survivors. Activated CD8+ T cells can also control virus-infected cells without destroying the cell. After hepatitis B infection in chimpanzees, CD8+ T cells secreted IFN-γ and TNF-α to halt viral replication and reduce viral loads in hepatocytes [30]. Alternatively, a study in influenza-infected cells showed that surviving cells upregulated the inhibitory ligand PD-L1 (programmed cell death ligand 1), which binds to PD-1 on CD8+ T cells, but they did not use this to evade CD8+ T cell killing [28]. Instead, surviving cells no longer presented IAV antigen and could not be detected by these cells, suggesting that survivor cells rapidly clear infection to evade mass killing from the adaptive immune system.

Further evidence of the key role of T cell-mediated immunity in POMV-infected cells was shown by our gene co-expression analysis. The topological structure of HEALTHY and MORIBUND networks regarding connections to differentially connected key regulators was significantly divergent. In the MORIBUND network, *RELA/p65* and *PRDM1* seemed to play a more central role than *HLF*, while the latter appeared to have a more dominant role in healthy fish. This is because a higher number of connections within the network indicates a tight coordination of gene expression, in which a small change in the expression of the central gene will rapidly influence all the other connected genes. Therefore, co-expressed genes are expected to work cooperatively for a specific function or pathway. Hepatic leukemia factor (HLF) regulates hematopoiesis and may protect hematopoietic stem cells from exhaustion triggered by stress responses from the immune system [31]. In addition, a recent study using a mouse model showed that the failure to downregulate *HLF* inhibited T cell development [32], suggesting that the downregulation and loss of connections of this transcription factor in moribund fish could also be related to the strong cellular response observed here during the infection with POMV. In contrast, *RELA* (p65 subunit) encodes for a member of the nuclear factor of kappa β (NF-κβ) family of transcription factors and has a critical role in the survival of activated effector CD8 T cells [33]. The *PRDM1* gene encodes for a transcriptional repressor (Blimp-1), which is a key regulator required for CD8+ T cells to differentiate into functional killer T cells. A study in mice showed that animals lacking this gene rapidly succumbed to infection with influenza virus [34]. Overall, the significant changes in the gene co-expression networks between the HEALTHY and MORIBUND groups explored in this study suggest an important role of these transcription factors in the molecular events that underpin POMV infection [9].

Altogether, our findings reiterate the power of functional genomics in untangling the complexities of host–pathogen interactions and viral pathogenesis. Despite the genomic divergence between POMV and other orthomyxoviruses, including ISAV, there is a remarkable similarity in the host response. As shown during the infection with highly pathogenic strains of ISAV [6,35,36] and pandemic influenza viruses [37,38], the transcriptome profiles of POMV-infected fish show a dramatic activation of the innate immune system and induction of pro-inflammatory cytokines, leading to severe tissue damage and morbidity. However, the subsequent activation of T cell-mediated immunity and reduced inflammation may be key factors contributing to survival and viral clearance.

## 4. Materials and Methods

### 4.1. Experimental Fish and Husbandry

Fish used in this study were all-female diploid Atlantic salmon smolts (mean ± SE weight: 119 ± 1 g) obtained from the Florentine hatchery (Salmon Enterprises of Tasmania, SALTAS). POMV has not been detected in freshwater fish in Tasmania, and therefore, the fish used for the challenge trials were considered specific (POMV) pathogen-free stock [3]. Fish had been vaccinated against yersiniosis by bath as fry (at 2.5 g and 8 g) and intraperitoneally (IP) at the parr stage (50 g). Prior to transfer, 15 fish from the cohort were independently sampled for disease testing. This included a bacterial culture of kidney on blood agar and the histopathology of major organs (liver, spleen, heart, gill, kidney, pyloric caecae, pancreas, stomach, eye, skin, and brain). No signs of disease were found in this assessment.

The experiment was conducted at the Biosecure Fish Facility (BFF) of the Fish health unit of the Department of Primary Industries, Water & Environment (DPIPWE) (Kings Meadows, Tasmania). During the challenge experiment, fish were placed in 1000 L tanks with seawater at full salinity (35 ppt) and 15 ℃ ± 1 in a recirculating aquaculture system. Un-ionized ammonia (NH_3_-N) was measured daily and kept below a safe limit for salmon post-smolts in seawater (<0.04 mg/L, Pennel and McLean, 1996; Fivelstad et al. 1995) by increasing the water exchange rates if needed. The measured pH was 7.8–7.9 in all tanks. All animal procedures were approved by DPIPWE’s Animal Ethics Committee (AEC) under AEC project number 3/2018-19.

### 4.2. POMV Challenge

Samples for this study were optained from the POMV challenge experiment described in detail in Samsing et al. [10]. In brief, Atlantic salmon post-smolts were challenged in seawater with POMV via cohabitation with trojan fish intraperitoneally injected (IP) with 200 µL of cell culture supernatant containing POMV at a titer of 10^8.8^ TCID_50_ mL^−1^ (tissue culture infective dose 50% per millilitre) [3]. The adipose fin of trojan fish was clipped to distinguish them from non-injected fish. Trojan fish were randomly allocated into four replicated cohabitation tanks (22 trojan fish per tank), and these were additionally stocked with 32 cohabitation fish. Nine randomly chosen fish were sampled from a holding tank before injection of the trojans and used as a pre-challenge control. Samples of moribund and surviving fish were taken from all tanks. Further details for each sample can be found in Supplementary Table S1.

The trial proceeded for 20 days post infection (dpi) of the trojan fish. During the trial, tanks were monitored continuously (three times daily at 8 h intervals), and moribund fish were immediately removed and euthanized with 100 mg L^−1^ of AQUI-S™ (Aqui-S, Lower Hutt, New Zealand) and sampled as described in the following section. Moribund fish were animals showing overt clinical signs of POMV [3] and were sampled during the peak of infection between 7 and 15 dpi. Moribund fish were found unresponsive but still swimming near the surface of the tank, and they presented dark coloration on their skin.

### 4.3. Sample Collection and Processing

Tissue samples from the liver, spleen, and head kidney were aseptically collected from pre-challenge control fish (n = 9), all moribunds (n = 21), and from a random subset of the survivors (n = 10). Tissue samples were not collected from fish found dead at the time of inspection. All samples for RNA extraction were collected in RNAlater^®^, stored at 4 ℃ overnight, and then placed at −80 ℃ until extraction. Tissue samples (liver, head kidney) for histology (stained with hematoxylin–eosin) and immunohistochemistry (IHC) were collected in 10% buffered formalin from control fish (n = 3) and a subset of the moribund fish (n = 5). Samples for IHC were processed using a polyclonal anti-POMV immune serum raised in rabbits [1] at 1:400. Liver and head kidney samples from the same fish (n = 3 control and n = 5 subset of moribunds) were collected for virus titration in bead homogenizer tubes (Lysing Matrix E 2 mL tubes, MP Biomedicals™) and stored at −80 ℃ until analysis. Samples for viral titration were seeded in Chinook salmon embryo (CHSE) cells and incubated at 15 ℃ in 2% CO_2_ for 21 days; then, wells were visually inspected for signs of characteristic POMV-like cytopathic effect [1]. The TCID_50_ was calculated according to the Reed and Muench TCID_50_ calculation method [39]. The processing of samples for IHC and viral titration is described in detail in Samsing et al. [10].

### 4.4. RNA Extraction and Sequencing

Tissue samples were weighed (≈20 mg) and placed into Lysing Matrix E 2 mL tubes (MP Biomedicals™, Santa Ana, CA, USA) for disruption and homogenization with RLT-Plus buffer/β-mercaptoethanol (600 μL) in a TissueLyser II (Qiagen, Venlo, Netherlands) for 2 min. The lysate was centrifuged at full speed, and the supernatant was used to extract total RNA using the RNeasy Plus Kit (Qiagen) as per the manufacturer’s instructions. Total RNA was eluted in a final volume of 40 µL of RNase-free water. From the RNA extracts, 24 samples were sent for the sequencing of messenger RNA (mRNA). These 24 samples were selected among the four experimental groups described in Section 4.6 (controls, POMV-positives, POMV-suspects, and survivors), randomly sampling three fish per group and collecting two different tissues (liver and head kidney) from each fish. Sequencing reads were produced as 100-bp paired-end reads across three lanes on a HiSeq 2000 sequencer (Illumina, San Diego, CA, USA) by Macrogen (Seoul, Korea) using the TruSeq stranded mRNA protocol.

### 4.5. Real-Time PCR

The real-time PCR assay that was used to detect POMV RNA in all samples was conducted following Mohr et al. [1]. RNA was extracted from tissues lysed in RLT buffer as described previously. The one-step TaqMan^®^ reverse transcriptase real-time PCR assay targeting segment 5 of the POMV genome [1] was performed on an Applied Biosystems 7500 Fast Instrument (Thermo Fisher Scientific, Waltham, MA, USA). Independent treatment replicates were analyzed in duplicate. The AgPath one-step RT-PCR (Thermo Fisher Scientific) chemistry was used for real-time PCR. The 25 μL reaction mixture was constituted by 5.75 μL of AgPath buffer, 12.5 μL RT-PCR enzyme mix, 1.25 μL forward and reverse primers (18 μM), 1.25 μL TaqMan probe (5 μM), and 2 μL of the template. The concentration of input RNA was estimated using ultraviolet (UV) absorbance in a NanoDrop^®^ spectrophotometer and normalized to 10 ng μL^−1^ for all samples. Cycling parameters were 10 min at 45 ℃, 10 min at 95 ℃, and then 40 cycles at 95 ℃ for 15 s and 60 ℃ for 45 s. As an internal positive control on each plate, all samples were analyzed for the expression of the housekeeping gene elongation factor 1 alpha, ELF1α [40].

### 4.6. Experimental Groups

Experimental groups were divided according to disease stage into control, moribund, and survivor fish. Control fish were randomly sampled from a holding tank before the injection of trojan fish. Moribund fish were those presenting the characteristic clinical signs of POMV pathology [3] including changes in behavior (loss of balance, lethargy), physical signs (dark coloration of the skin, petechiae of ventral areas of the body), and gross pathological findings at the necropsy (mucus in the gut, generalized congestion, petechial hemorrhages in visceral fat and splenomegaly). Survivor fish were those still alive on day 20 post-challenge, when the experiment was terminated after the morbidity rate had reached a plateau phase [10] and presented no overt clinical signs of disease.

Moribund fish were further subdivided into POMV-positive and POMV-suspects based on the results of the real-time PCR. POMV-positive moribunds were those with a cycle threshold (CT) value < 38 in both tissues (liver and head kidney), and POMV-suspect moribunds were those with either no CT or a CT > 38. Results mainly focus on the response of confirmed POMV-positive fish—those that tested positive to the qPCR—and survivors.

### 4.7. Read Mapping

FastQC v.0.11.5 was used to assess the quality of forward and reverse raw read files, granting a PASS status for most fastQC statistics. Then, STAR (v.2.5.3a) was used to map paired-end reads to the most recent assembly of the Atlantic salmon genome (ICSASG_v2; GenBank Accession No. GCF_000233375.1) [41]. Mapping parameters included a maximum of 2 mismatched nucleotides and 20 multiple alignments per read; if exceeded, the read was considered unmapped. Uniquely mapped paired reads were counted and assigned to genes (NCBI S. salar Annotation Release 100) using FeatureCounts (SourceForge Subread package v.1.6.2). Only reads with both ends mapped to the same gene were considered in downstream analyses.

For each sample, forward and reverse reads were also mapped against the POMV genome isolate POMV14-01514 (GenBank Accession Numbers MN241407-MN241414) using Bowtie2 v.2.2.9. SAMtools v.0.1.19 was used to convert the resulting SAM files to BAM files. Segment coverage of the POMV genome was quantified by converting sorted and indexed BAM files into BED files and then using the genomeCoverageBed command in BEDtools v.2.26.0. The POMV per base coverage was normalized by the proportion of viral reads among the total reads in each sample. Then, mean normalized per base coverage was calculated for each viral segment expressed in each tissue and disease stage.

### 4.8. Differential Expression and Functional Enrichment Analyses

Differential expression (DE) and functional enrichment analyses were performed in R v.3.5.1 (R Core Team, 2018). Differential expression was estimated from gene count data using R/Bioconductor’s package Limma v.3.36.5. Firstly, a filtering step retained all raw reads that represented at least 0.5 counts per million in three samples. The filtered raw count matrix was normalized to account for differences in library sizes using the trimmed mean of M-values (TMM) method implemented in EdgeR v.3.26.7 [42]. Subsequently, the mean–variance relationship of the log2 counts was estimated using the voom method (Limma package), which generates a precision weight for each observation and enters these into the limma empirical Bayes analysis [43]. Two linear models were fit to the data: an additive two-factor model with disease stage (POMV-positive, POMV-suspects, and survivors) and tissue type (liver and head kidney), and a nested interaction model with tissue type within disease stage (only POMV-positives and survivors) to assess the effect of tissue type on differential expression in each disease stage. Both models also incorporated the tank from which fish were sampled as a random effect or blocking factor using the ‘duplicateCorrelation()’ function in the Limma package. After model fitting, pair-wise contrasts were performed between disease stages and control groups. Gene transcripts with an absolute log2 fold change (logFC) > 2 and false discovery rate (FDR) adjusted *p*-value (Padj) < 0.05 (Benjamini–Hochberg correction) were considered differentially expressed for functional and pathway enrichment analyses. For survivors, we used an absolute logFC > 2, but a Padj < 0.1. The clustering of treatment groups and identification of potential outliers was examined using multi-dimensional scaling (MDS). The MDS plot was created using the plotMDS function implemented in the Limma package and is included in supplementary Material (Figure S1).

Atlantic salmon official gene identifiers (Entrez gene IDs) for all differentially expressed genes were retrieved using R/Bioconductor AnnotationHub (snapshot date: 02/05/2019) package v. 2.14.2. Enrichment analyses (Gene Ontology and KEGG) were conducted on ranked upregulated and downregulated gene lists using Entrez gene IDs in clusterProfiler in R v.3.10.1. Background gene lists were compiled by retrieving all gene identifiers from all genes expressed in at least three samples (n = 33,741). Gene Ontology (GO) and KEGG terms with Padj < 0.05 were considered significantly enriched by differentially expressed gene sets.

### 4.9. Gene Co-Expression Network Construction

Network construction was based on a normalized gene expression matrix in a log2 scale obtained using the ‘rlog’ function in DEseq2 [44]. Relevant genes for network construction were defined based on the following criteria:

**(A) Differentially expressed genes**: Differentially expressed (DE) genes were filtered by an absolute log2 FC > 2, Padj < 0.05 and a normalized mean expression (NME) > 1.39. These DE genes were obtained using the limma–voom pipeline as described before (in Section 4.8), and they came from three contrasts: control vs. POMV-positive fish (n = 852), control vs. POMV-survivors (n = 384), and survivors vs. POMV-positives (n = 385). Genes from the control vs. survivor contrast were only filtered by Padj < 0.05 to keep a longer list of genes.

**(B) Key regulatory transcription factors**: A list of 3552 Atlantic salmon transcription factors obtained from Mohamed et al. [45] was compiled to identify key regulatory genes for inclusion in the co-expression network. Transcription factors were compared to potential target genes composed of the differentially expressed genes from each contrast, using the regulatory impact factor metrics [46]. This analysis includes two metrics designed to assign scores to regulator genes consistently differentially co-expressed with target genes and to those with the most altered ability to predict the abundance of target genes. The contrast defined here was healthy vs. moribunds, independently of the tissue. Significant scores were those deviating ± 1.96 standard deviation from the mean (corresponding to *p* < 0.05). Genes with mean expression values lower than the mean of all genes expressed were not considered in this analysis.

Nodes in the co-expression networks included the genes selected in the two categories described previously, and significant connections (or edges in the network) were identified using the Partial Correlation and Information Theory (PCIT) algorithm [47]. PCIT determines the significance of the correlation between two nodes after accounting for all the other nodes in the network. Three networks were generated based on the same genes, namely: (1) ALL—considering all samples and tissues (n = 24), (2) HEALTHY—considering controls and survivors samples from both tissues (n = 12), and (3) MORIBUND, considering POMV-suspects and POMV-positives (n = 12). Edges or connections between gene nodes were included when the partial correlation coefficient was greater than two standard deviations from the mean (*p* < 0.01). Cytoscape version 3.7.0. was used to visualize the output of the PCIT.

### 4.10. Differential Connectivity

To explore differential connectivity between “clinically healthy” and “clinically affected” or moribund fish, we compared the HEALTHY and MORIBUND networks mentioned previously, which were constructed using the same genes as the ALL network and the same methodology. The number of connections for each gene in each condition was computed and scaled so that connectivity ranged between 0 and 1, making it possible to compare the same gene between the two networks. The connectivity in the MORIBUND network was subtracted from the connectivity in the HEALTHY network, and results deviating ± 1.96 standard deviations from the mean were considered significant.

## Data Availability

RNA-seq data has been submitted to the NCBI’s Sequence Read Archive and will be released at the time of publication. BioProject ID PRJNA659597 metadata is available at: https://dataview.ncbi.nlm.nih.gov/object/PRJNA659597?reviewer=sodlnl2a3r4rbqrufmb21c1s8a

## Supplementary Materials

The following are available online at https://www.mdpi.com/2076-0817/9/10/807/s1, Figure S1: Multi-dimensional scaling (MDS) performed on normalized expression values to visualize the clustering of replicates and treatment conditions, Figure S2: Expression of POMV genomic segments in moribund fish mapping RNA-seq raw reads against the POMV genome in liver (a) and head kidney (b), Figure S3: Annotated gene co-expression network for panel (a) in Figure 8, Figure S4: Annotated gene co-expression network for panel (b) in Figure 8. Table S1: Detection of POMV by RNAseq reads mapped to the POMV genome (total raw reads), RT-qPCR and immunohistochemistry, Table S2: Summary statistics of number of RNAseq reads obtained and mapped to the Atlantic salmon (*Salmo salar*) genome, Tables S3 and S4: Tables of differentially expressed genes in different conditions, Table S5: Functional enrichment analysis for all conditions.
