# Supplementary material for: Transcriptome Response of Atlantic Salmon (Salmo salar) to a New Piscine Orthomyxovirus
Source: Pathogens. 2020 Sep 30;9(10):807. doi: 10.3390/pathogens9100807 (PMC7600774; doi:10.3390/pathogens9100807)
Supplement: Supplementary file 1 [file pathogens-09-00807-s001.zip › Supplementary_material_M2/Suppl_Figure_Legends.docx]

Transcriptome response of Atlantic salmon (*Salmo salar*) to a new piscine orthomyxovirus

Francisca Samsing ^1,^*, Pamela Alexandre ^2^, Megan Rigby ^1^, Richard S. Taylor^1^, Roger Chong^2^ and James W. Wynne ^1,^*

^1^ CSIRO Agriculture and Food, Hobart, TAS, Australia; Francisca.samsingpedrals@csiro.au

^2^ CSIRO Agriculture and Food, Brisbane, QLD, Australia; e-mail@e-mail.com

***** Correspondence: F.S.: [Francisca.samsingpedrals@csiro.au](mailto:Francisca.samsingpedrals@csiro.au), J.W.W.: [james.wynne@csiro.au](mailto:james.wynne@csiro.au)

**Supplementary Figure Legends**

**Figure S1.** Multi-dimensional scaling (MDS) performed on normalized expression values to visualize the clustering of replicates and treatment conditions. TMM (trimmed mean of M-values) normalization was performed to eliminate composition biases between libraries.

**Figure S2.** Expression of POMV genomic segments in moribund fish (POMV-positives and POMV-suspects) mapping RNA-seq raw reads against the POMV genome. Read depth was calculated as mean per-base coverage, normalized by the proportion of viral reads among total reads in each sample. Expression patterns are shown for (**a**) head kidney and (**b**) liver.

**Figure S3:** Annotated gene co-expression network for panel (**a**) in Figure 8. This HEALTHY network considers the first neighbours (direct connections) of the only three transcription factors or key regulators that were also differentially expressed genes and differentially connected by comparing the HEALTHY and MORIBUND networks.

**Figure S4:** Annotated gene co-expression network for panel (**b**) in Figure 8. This MORIBUND network considers the first neighbours (direct connections) of the only three transcription factors or key regulators that were also differentially expressed genes and differentially connected by comparing the HEALTHY and MORIBUND networks.
