# Supplementary figures and images for: Transcriptome Response of Atlantic Salmon (Salmo salar) to a New Piscine Orthomyxovirus
Source: Pathogens. 2020 Sep 30;9(10):807. doi: 10.3390/pathogens9100807 (PMC7600774; doi:10.3390/pathogens9100807)

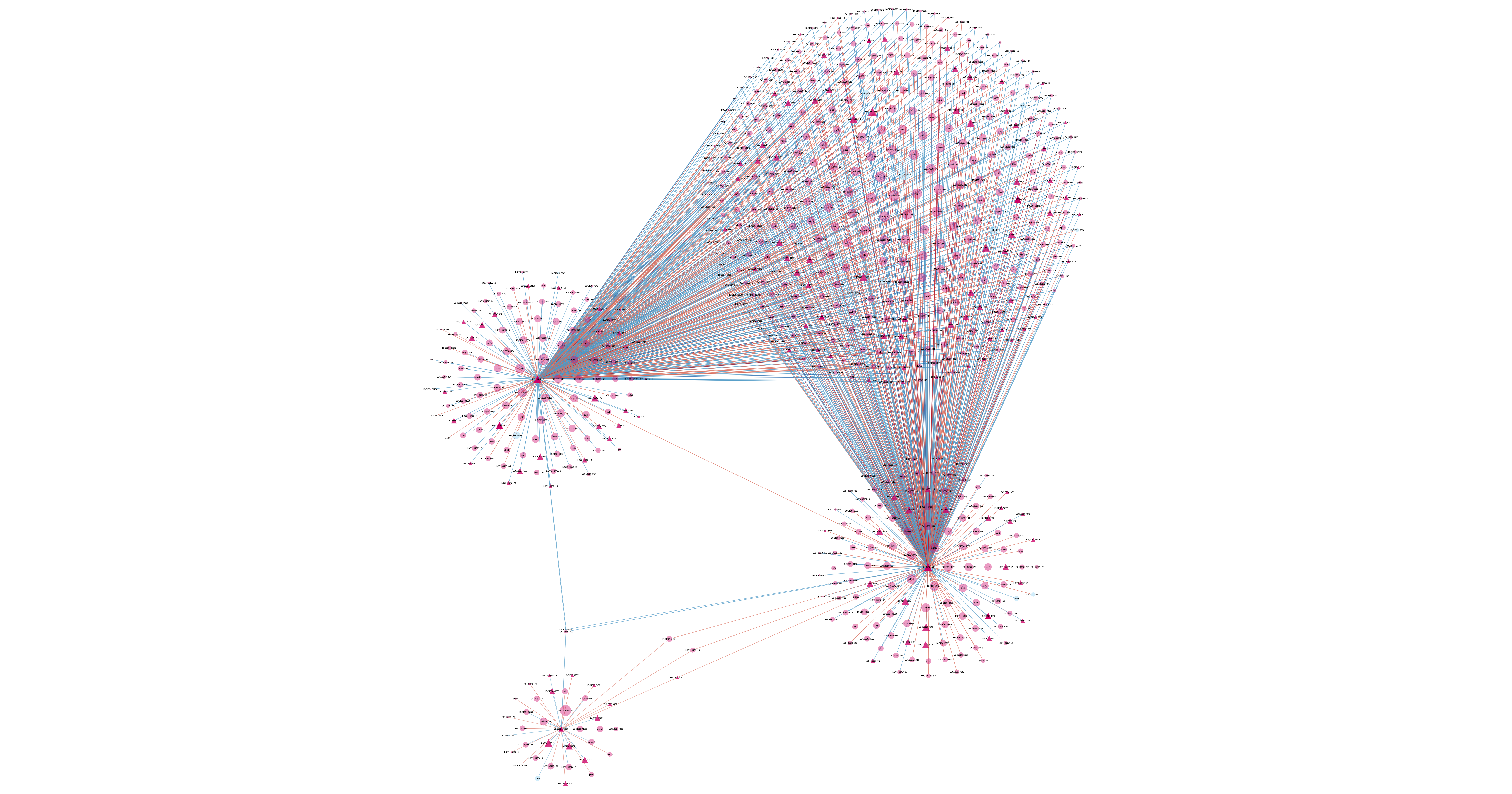

Supplement: Supplementary file 1 [file pathogens-09-00807-s001.zip › Supplementary_material_M2/Supplemenatary_Figure_S4_3DETFDiffConn_moribund.png]

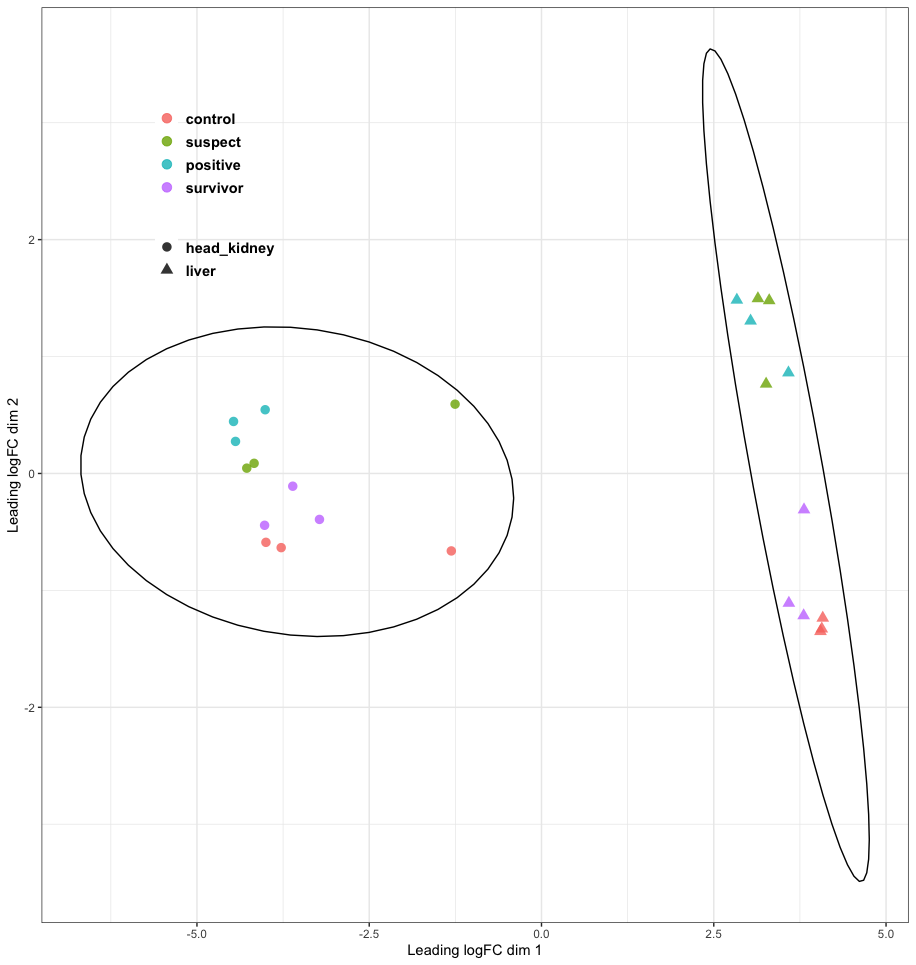

Supplement: Supplementary file 1 [file pathogens-09-00807-s001.zip › Supplementary_material_M2/Supplemenatary_Figure_S1_MDS_plot.png]

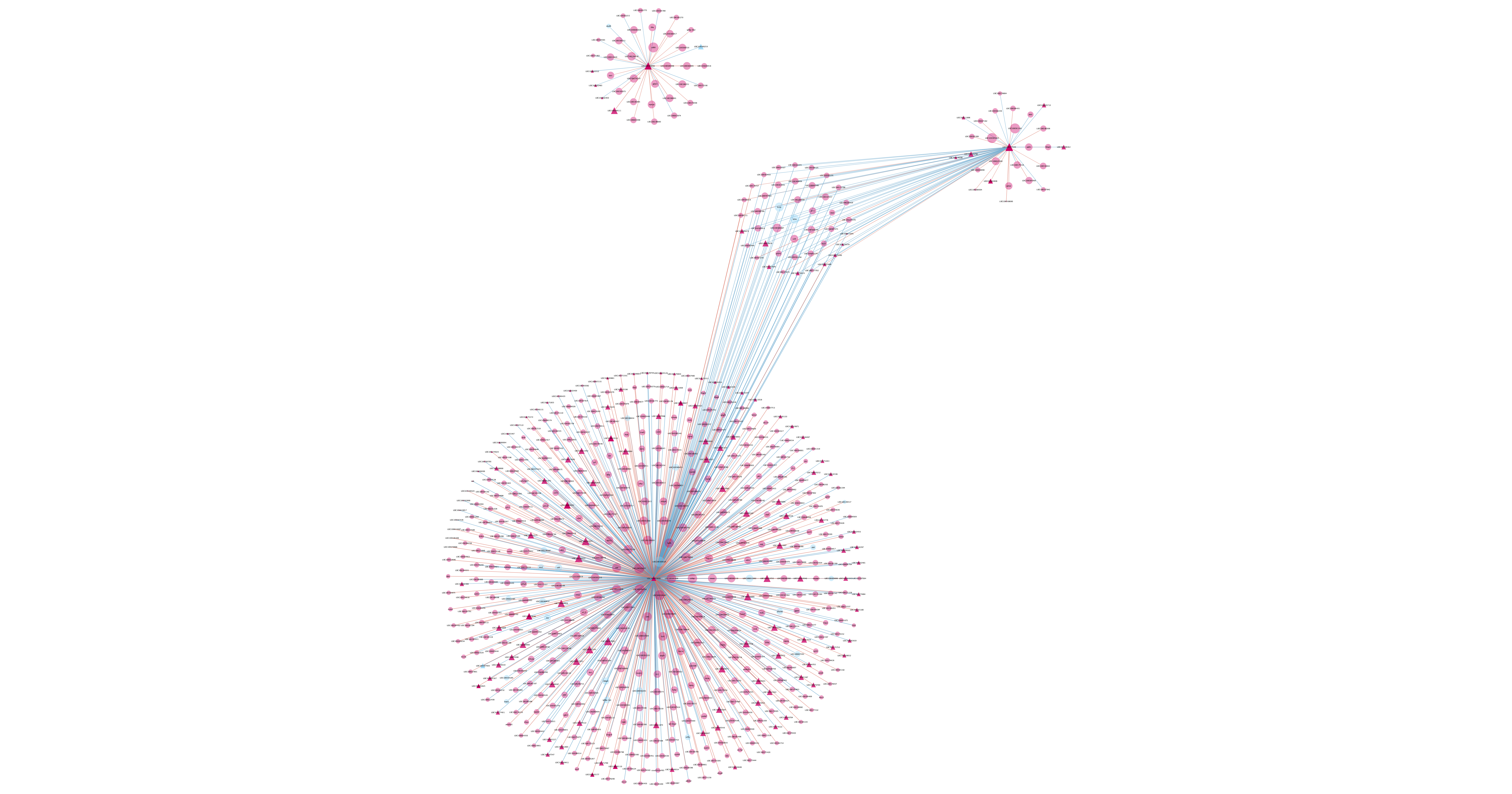

Supplement: Supplementary file 1 [file pathogens-09-00807-s001.zip › Supplementary_material_M2/Supplemenatary_Figure_S3_3DETFDiffConn_healthy.png]

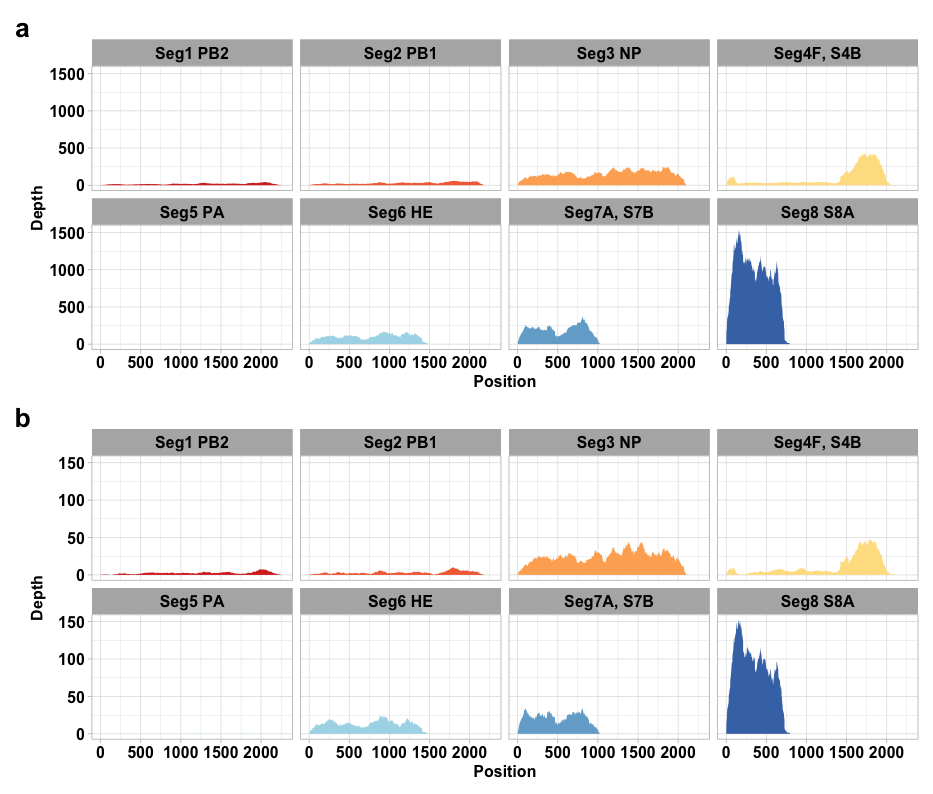

Supplement: Supplementary file 1 [file pathogens-09-00807-s001.zip › Supplementary_material_M2/Supplemenatary_Figure_S2_POMV_reads_HK_liver.png]
